# Assessment of hypercholesterolemia prevalence and its demographic variations in the Republic of Kazakhstan

**DOI:** 10.1038/s41598-024-57884-4

**Published:** 2024-03-26

**Authors:** Mukhtar Kulimbet, Kairat Davletov, Timur Saliev, Dimash Davletov, Berik Dzhumabekov, Bauyrzhan Umbayev, Aigerim Balabayeva, Shynar Tanabayeva, Ildar Fakhradiyev

**Affiliations:** 1 S.D. Asfendiyarov Kazakh National Medical University, 94, Tole-bi Str., 050020 Almaty, Republic of Kazakhstan; 2 Al-Farabi Kazakh National University, Almaty, Republic of Kazakhstan; 3 National Laboratory Astana, Center for Life Sciences, Nazarbayev University, Astana, Republic of Kazakhstan

**Keywords:** Hypercholesterolemia, Prevalence, Cholesterol levels, Kazakhstan, Diseases, Risk factors

## Abstract

Hypercholesterolemia is a major risk factor for cardiovascular disease, the leading cause of death in Kazakhstan. Understanding its prevalence is vital for effective public health planning and interventions. This study aimed to assess the scale of hypercholesterolemia in the Republic of Kazakhstan and to identify differences among distinct population groups. A cross-sectional study involving 6720 participants (a nationally representative survey.) aged 18–69 was conducted from October 2021 to May 2022 across all 17 regions of Kazakhstan. The magnitude of hypercholesterolemia was 43.5%. Cholesterol levels were determined through blood biochemical analysis. Age, sex, geographic location, and ethnicity served as covariates. The majority of participants (65.49%) were from urban areas with an almost equal gender distribution (50.07% male and 49.93% female). The predominant age groups were 18–29 years (25.71%) and 30–39 years (25.12%), and 65.09% identified as Kazakh. The prevalence increased with age, with the 60–69 age group showing the highest rate at 71.14%. Women had slightly higher rates than men. Geographical differences were evident, with regions like Astana city and Almaty region showing significant disparities. Kazakhs had a lower rate compared to other ethnicities. Age, region, and BMI were significant predictors for hypercholesterolemia in both binary and multivariate logistic regression analyses. The study revealed a significant prevalence of hypercholesterolemia in Kazakhstan, with increasing age as a major determinant. Women, especially those over 50, and certain regions showed higher cholesterol levels. The disparities observed across regions and ethnicities suggest the need for targeted public health interventions to address this pressing health concern.

## Introduction

In today's global health landscape, cardiovascular diseases (CVDs) have emerged as one of the leading causes of mortality worldwide^1^, as well as in the Republic of Kazakhstan^2^. One major contributor to the development of these conditions is hypercholesterolemia—the condition of having elevated levels of cholesterol in the blood^3^. A substantial body of research, including numerous clinical and epidemiological studies, points towards a compelling association between dyslipidemia—abnormalities in lipid metabolism, and the development of atherosclerosis, a disease characterized by the build-up of plaques in the arteries^4^. Particularly, hypercholesterolemia, alongside arterial hypertension and tobacco smoking, has been recognized as one of the top three powerful risk factors for CVDs^5^.

Moreover, the impact of age as a risk factor is undeniably significant. The aging process is known to influence lipid metabolism, potentially leading to increased cholesterol levels and altered lipid profiles, thus heightening cardiovascular risk^6^.

Despite this recognized burden, comprehensive data on hypercholesterolemia's prevalence and its demographic disparities within the Kazakhstani population remain sparse^7,8^, underlining the need for focused research in this area.

In light of this evidence, it is increasingly clear that the reduction of blood cholesterol levels should be regarded as a pivotal approach in the preventative strategies against CVDs^9^.

Given this background, the present study is devoted to an in-depth examination of the cholesterol levels among the population of Kazakhstan, with a special focus on the predisposition to hypercholesterolemia in different demographic groups.

The interpretation and analysis of such data is of paramount importance when it comes to the planning and execution of preventative measures against CVDs at the national scale^10^. Accurately identifying high-risk groups can streamline the allocation of healthcare resources, ensuring that interventions are as efficient and impactful as possible^11^.

In this study, we aspire to do more than simply assess the scope of hypercholesterolemia in Kazakhstan. We also aim to unearth any potential disparities that exist between different demographic groups. The insights gained from our research can then be utilized in the development of targeted public health programs, contributing to the overall improvement of population health and prevention of CVDs.

## Results

From a total of 6720 study participants, 2971 (43.51%) were found to have total cholesterol levels > 5 mmol/l. The study's demographic distribution comprised participants predominantly from urban areas (65.49%), with a nearly equal gender distribution (50.07% male, 49.93% female) (Table 1). A significant percentage of the participants were aged between 18 and 29 years (25.71%) and 30–39 years (25.12%). The majority identified as Kazakh (65.09%), followed by Russian (23.07%). Mean values of total cholesterol was 4.93 ± 1.15.

**Table 1:** Demographic characteristics of the participants.

Characteristics	Count	Percent
Age		
18–29	1728	25.71
30–39	1688	25.12
40–49	1310	19.49
50–59	1159	17.25
60–69	835	12.43
Gender		
Male	3365	50.07
Female	3355	49.93
Place of residence		
Urban	4401	65.49
Rural	2319	34.51
Region		
Astana city	448	6.67
Almaty city	560	8.33
Akmola region	336	5.00
Aktobe region	336	5.00
Almaty region	560	8.33
Atyrau region	336	5.00
West Kazakhstan region	224	3.33
Zhambyl region	448	6.67
Karaganda region	448	6.67
Kostanai region	336	5.00
Kyzylorda region	336	5.00
Mangistau region	336	5.00
Turkestan region	560	8.33
Pavlodar region	336	5.00
North Kazakhstan region	224	3.33
East Kazakhstan region	448	6.67
Shymkent city	448	6.67
Ethnicity		
Kazakh	4374	65.09
Russian	1550	23.07
Others	796	11.85
Education		
No education	85	1.26
High school (9th grade)	416	6.19
High school (11th grade)	1797	26.74
Bachelor	3099	46.12
Graduate	1323	19.69
Marital status		
Single	1527	22.72
Married	4447	66.18
Married, live separate	63	0.94
Divorced	386	5.74
Widow	225	3.35
Civil marriage	72	1
Smoking		
Yes	1284	19.11
No	5436	80.89
BMI		
Normal weight (< 25)	2858	42.53
Overweight (≥ 25)	3862	57.47
Total cholesterol (mmol/l)	4.93*	1.15**

*Mean; **Standard deviation.

Regarding hypercholesterolemia status (Table 2), age was a significant factor, with older age groups showing a higher prevalence. The 60–69 age group had the highest hypercholesterolemia rate at 71.14%, followed by the 50–59 age group at 67.04%. The Chi-square value for age indicated statistical significance (*P* < 0.0001). Gender and place of residence also showed significant associations with hypercholesterolemia, with females and urban residents having slightly higher rates (*P* = 0.0480 and *P* = 0.0001 respectively). Region-wise, significant variations were observed, with Almaty city and Almaty region being notable examples. Ethnicity also played a role, with Kazakhs having a reduced hypercholesterolemia rate compared to “Others” (*P* < 0.0001). Marital status indicated single individuals having the lowest rate of hypercholesterolemia.

**Table 2:** Univariate analysis regarding HC status.

Variables	Hypercholesterolemia		Total N (%)	Chi-square (X^2^)	*P* value
	No (n =) n (%)	Yes (n =) n (%)			
Total	3796 (56.49)	2924 (43.51)	6720 (100)		
Age					
18–29	1423 (37.49)	305 (10.43)	1728 (25.71)	1056.4	< 0.0001
30–39	1091 (28.74)	597 (20.42)	1688 (25.12)		
40–49	659 (17.36)	651 (22.26)	1310 (19.49)		
50–59	382 (10.06)	777 (26.57)	1159 (17.25)		
60–69	241 (6.35)	594 (20.31)	835 (12.43)		
Gender					
Male	1941 (51.13)	1424 (48.70)	3365 (50.07)	3.9	0.0480
Female	1855 (48.87)	1500 (51.30)	3355 (49.93)		
Place of residence					
Urban	2560 (67.44)	1841 (62.96)	4401 (65.49)	14.6	0.0001
Rural	1236 (32.56)	1083 (37.04)	2319 (34.51)		
Region					
Astana city	296 (7.8)	152 (5.2)	448 (6.67)	83.8	< 0.0001
Almaty city	306 (8.06)	254 (8.69)	560 (8.33)		
Akmola region	191 (5.03)	145 (4.96)	336 (5.00)		
Aktobe region	194 (5.11)	142 (4.86)	336 (5.00)		
Almaty region	291 (7.67)	269 (9.2)	560 (8.33)		
Atyrau region	176 (4.64)	160 (5.47)	336 (5.00)		
West Kazakhstan region	121 (3.19)	103 (3.52)	224 (3.33)		
Zhambyl region	229 (6.03)	219 (7.49)	448 (6.67)		
Karaganda region	245 (6.45)	203 (6.94)	448 (6.67)		
Kostanai region	189 (4.98)	147 (5.03)	336 (5.00)		
Kyzylorda region	220 (5.8)	116 (3.97)	336 (5.00)		
Mangistau region	161 (4.24)	175 (5.98)	336 (5.00)		
Turkestan region	365 (9.62)	195 (6.67)	560 (8.33)		
Pavlodar region	177 (4.66)	159 (5.44)	336 (5.00)		
North Kazakhstan region	105 (2.77)	119 (4.07)	224 (3.33)		
East Kazakhstan region	256 (6.74)	192 (6.57)	448 (6.67)		
Shymkent city	274 (7.22)	174 (5.95)	448 (6.67)		
Ethnicity					
Kazakh	2638 (69.49)	1736 (59.37)	4374 (65.09)	77.0	< 0.0001
Russian	747 (19.68)	803 (27.46)	1550 (23.07)		
Others	411 (10.83)	385 (13.17)	796 (11.85)		
Education					
No education	48 (1.2)	37 (1.27)	85 (1.26)		
High school (9th grade)	212 (5.58)	204 (6.98)	416 (6.19)		
High school (11th grade)	1024 (26.98)	773 (26.44)	1797 (26.74)		
Bachelor	1704 (44.89)	1395 (47.71)	3099 (46.12)		
Graduate	808 (21.29)	515 (17.61)	1323 (19.69)		
Marital status					
Single	1118 (29.45)	409 (13.99)	1527 (22.72)	280.9	< 0.0001
Married	2366 (62.33)	2081 (71.17)	4447 (66.18)		
Married, live separate	32 (0.84)	31 (1.06)	63 (0.94)		
Divorced	182 (4.79)	204 (6.98)	386 (5.74)		
Widow	65 (1.71)	160 (5.47)	225 (3.35)		
Civil marriage	33 (0.87)	39 (1.33)	72 (1)		
Smoking					
Yes	755 (19.89)	529 (18.09)	1284 (19.11)	3.4	0.0631
No	3041 (80.11)	2395 (81.91)	5436 (80.89)		
BMI					
Normal weight (< 25)	1948 (51.32)	910 (31.12)	2858 (42.53)	275.6	< 0.0001
Overweight (≥ 25)	1848 (48.68)	2014 (68.88)	3862 (57.47)		

**Figure 1:**
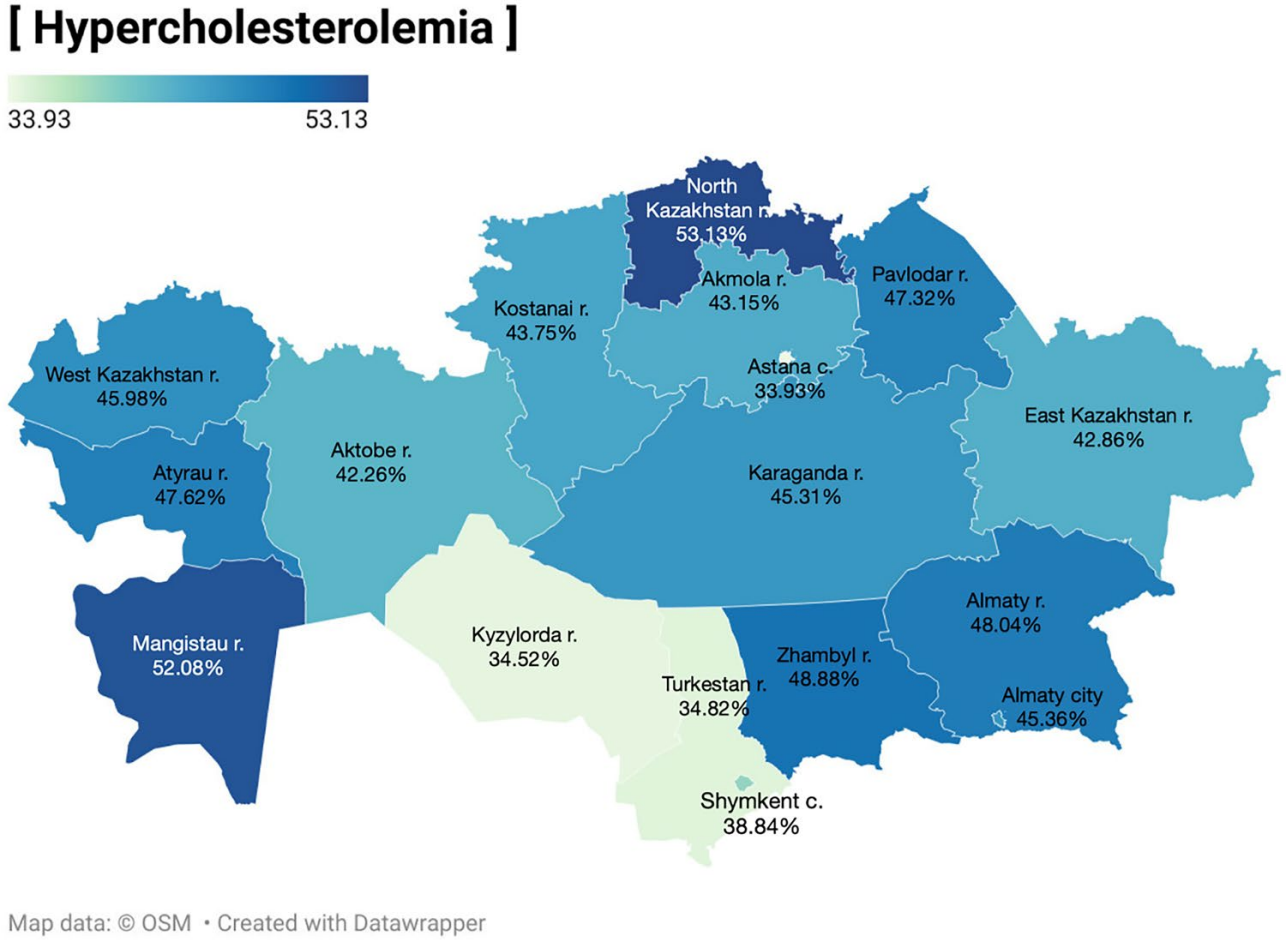
Prevalence of hypercholesterolemia throughout Kazakhstan (created via Datawrapper; available at https://www.datawrapper.de/maps). This figure illustrates the prevalence of hypercholesterolemia across various regions of Kazakhstan, based on the findings from a cross-sectional study conducted from October 2021 to May 2022. Each region’s prevalence rate is represented, providing insight into geographical variations within the country.

In the binary logistic regression results (Table 3), age remained a significant predictor of hypercholesterolemia, with older age groups having higher odds ratios. Gender differences were not statistically significant in this analysis. Region (Fig. 1) and ethnicity also showed significant variations, with some regions like Astana city having lower odds ratios. In terms of BMI, being overweight significantly increased the risk of hypercholesterolemia (OR = 2.33, *P* < 0.0001).

**Table 3:** Binary logistic regression of the influencing factors for hypercholesterolemia.

Variables	Hypercholesterolemia (%)	*P*-value	OR	95% CI
Age				
18–29*	17.65	–	–	–
30–39	35.37	< .0001	2.55	2.18–2.99
40–49	49.69	< .0001	4.61	3.91–5.43
50–59	67.04	< .0001	9.49	7.97–11.29
60–69	71.14	< .0001	11.50	9.47–13.96
Gender				
Male*	42.32	–	–	–
Female	44.71	0.0481	1.10	1.01–1.21
Place of residence				
Rural*	46.70	–	–	–
Urban	41.83	0.0001	0.82	0.72–0.90
Region				
Almaty region*	48.04	–	–	–
Astana city	33.93	< .0001	0.56	0.43–0.72
Almaty city	45.36	0.369	0.90	0.71–1.14
Akmola region	43.15	0.1562	0.82	0.63–1.08
Aktobe region	42.26	0.0934	0.79	0.6–1.04
Atyrau region	47.62	0.9038	0.98	0.75–1.29
West Kazakhstan region	45.98	0.603	0.92	0.68–1.26
Zhambyl region	48.88	0.7889	1.04	0.81–1.33
Karaganda region	45.31	0.3893	0.90	0.7–1.15
Kostanai region	43.75	0.2132	0.84	0.64–1.1
Kyzylorda region	34.52	< .0001	0.57	0.43–0.75
Mangistau region	52.08	0.2409	1.18	0.9–1.54
Turkestan region	34.82	< .0001	0.58	0.46–0.74
Pavlodar region	47.32	0.8358	0.97	0.74–1.27
North Kazakhstan region	53.13	0.1982	1.23	0.9–1.67
East Kazakhstan region	42.86	0.1012	0.81	0.63–1.04
Shymkent city	38.84	0.0035	0.69	0.53–0.88
Ethnicity				
Others	48.37	–	–	–
Kazakh	39.69	< .0001	0.70	0.60–0.81
Russian	51.81	0.1147	1.15	0.97–1.36
Education				
Graduate	38.93	–	–	–
No education	43.53	0.4001	1.21	0.78–1.88
High school (9th grade)	49.04	0.0003	1.51	1.21–1.88
High school (11th grade)	43.02	0.0219	1.18	1.03–1.37
Bachelor	45.01	0.0002	1.28	1.13–1.47
Marital status				
Single	26.78			
Married	46.80	< .0001	2.40	2.12–2.73
Married, live separate	49.21	0.0002	2.65	1.6–4.4
Divorced	52.85	< .0001	3.06	2.44–3.86
Widow	71.11	< .0001	6.73	4.94–9.17
Civil marriage	54.17	< .0001	3.23	2.01–5.21
Smoking				
Yes	41.20	–	–	–
No	44.06	0.0632	1.12	0.99–1.27
BMI				
Normal weight (< 25)	31.84	–	–	–
Overweight (≥ 25)	52.15	< .0001	2.33	2.11–2.58

*reference; %, percent; OR, Odds ratio; CI, Confidence intervals;

The multivariate logistic regression analysis (Table 4) revealed significant predictors of hypercholesterolemia. Age exhibited a consistent association, moreover, regional disparities were evident, with certain regions demonstrating statistically lower odds ratios for hypercholesterolemia compared to the reference region. Notably, BMI emerged as a robust predictor, indicating a significant increase in the risk of hypercholesterolemia among overweight individuals. While gender initially demonstrated a significant association in the univariate analysis. Similarly, place of residence and smoking status did not exhibit statistically significant associations with hypercholesterolemia in the multivariate model. Conversely, ethnicity, particularly Kazakh ethnicity, retained its significance, with Kazakh individuals displaying lower odds ratios for hypercholesterolemia compared to individuals from other ethnic backgrounds.

**Table 4:** Multivariate logistic regression of the influencing factors for hypercholesterolemia.

Variables	Hypercholesterolemia (%)	*P*-value	AOR	95% CI
Age				
18–29*	17.65	–	–	–
30–39	35.37	< .0001	2.29	1.92–2.73
40–49	49.69	< .0001	3.99	3.31–4.8
50–59	67.04	< .0001	8.19	6.72–9.98
60–69	71.14	< .0001	9.40	7.52–11.74
Gender				
Male*	42.32	–	–	–
Female	44.71	0.6049	1.03	0.92–1.16
Place of residence				
Rural*	46.70	–	–	–
Urban	41.83	0.1255	1.11	0.97–1.27
Region				
Almaty region*	48.04	–	–	–
Astana city	33.93	< .0001	0.54	0.4–0.74
Almaty city	45.36	0.2	0.84	0.64–1.1
Akmola region	43.15	0.0023	0.62	0.46–0.84
Aktobe region	42.26	0.1712	0.81	0.6–1.1
Atyrau region	47.62	0.4976	1.11	0.82–1.51
West Kazakhstan region	45.98	0.3792	0.85	0.6–1.21
Zhambyl region	48.88	0.7105	1.05	0.8–1.39
Karaganda region	45.31	0.0239	0.72	0.54–0.96
Kostanai region	43.75	0.0096	0.66	0.49–0.91
Kyzylorda region	34.52	0.0005	0.58	0.42–0.79
Mangistau region	52.08	0.029	1.40	1.04–1.9
Turkestan region	34.82	0.0002	0.60	0.46–0.79
Pavlodar region	47.32	0.1124	0.78	0.57–1.06
North Kazakhstan region	53.13	0.7764	0.95	0.67–1.35
East Kazakhstan region	42.86	0.003	0.65	0.49–0.86
Shymkent city	38.84	0.0771	0.77	0.57–1.03
Ethnicity				
Others	48.37	–	–	–
Kazakh	39.69	** < .**0001	0.69	0.58–0.82
Russian	51.81	0.717	0.97	0.8–1.17
Education				
Graduate	38.93	–	–	–
No education	43.53	0.4923	1.19	0.73–1.95
High school (9th grade)	49.04	0.7236	0.95	0.73–1.24
High school (11th grade)	43.02	0.0241	0.81	0.68–0.97
Bachelor	45.01	0.6223	1.04	0.89–1.22
Marital status				
Single	26.78			
Married	46.80	0.6526	1.04	0.89–1.21
Married, live separate	49.21	0.4598	1.24	0.7–2.18
Divorced	52.85	0.2214	1.18	0.91–1.53
Widow	71.11	0.0855	1.36	0.96–1.92
Civil marriage	54.17	0.1349	1.50	0.88–2.53
Smoking				
Yes	41.20	–	–	–
No	44.06	0.5294	1.05	0.91–1.21
BMI				
Normal weight (< 25)	31.84	–	–	–
Overweight (≥ 25)	52.15	< .0001	1.44	1.29–1.62

*reference; %, percent; AOR, adjusted odds ratio; CI, confidence intervals;

## Discussion

The findings from this comprehensive, cross-sectional study illuminate the widespread prevalence and demographic variations of hypercholesterolemia within the Republic of Kazakhstan, highlighting the imperative for immediate preventive measures and targeted interventions to curtail cardiovascular risks.

The significant prevalence of hypercholesterolemia identified through our investigation emphasizes a substantial public health concern, especially in light of the well-established correlation between elevated cholesterol levels and the development of cardiovascular diseases, which remain a leading cause of global morbidity and mortality^12^.

Our results affirm a progressive escalation in cholesterol levels and the frequency of hypercholesterolemia concomitant with advancing age, a pattern consistent with findings from previous global research endeavors^13^. The statistically significant association between age and blood cholesterol levels elucidates a profound biological nexus between age-related physiological changes and lipid metabolism. It is pivotal to acknowledge that age-related alterations in lipid profiles manifest distinctly between males and females, likely attributable to hormonal influences^14^. The underlying mechanisms for these changes encompass a decline in estrogen production, which during the reproductive years, contributes to maintaining a more favorable lipid profile, thereby safeguarding the vascular endothelium against atherosclerotic modifications^15^. This gender-specific disparity underscores the critical need for gender-sensitive approaches in the management and prevention of hypercholesterolemia, particularly addressing the hormonal transition periods such as menopause in women^16^. Consequently, these insights call for an integrated health strategy that not only focuses on the broader population but also considers the unique physiological and hormonal transitions experienced by women, to effectively mitigate the heightened cardiovascular risk associated with hypercholesterolemia.

The regional disparities in the prevalence of hypercholesterolemia within the Republic of Kazakhstan can be attributed to a confluence of environmental, socio-economic, and cultural factors^17^, that influence lifestyle, dietary habits, the availability and quality of healthcare services, as well as the education level and overall health of the population. These findings underscore the imperative for a regional approach in the design and implementation of preventive programs and interventions aimed at reducing cholesterol levels within the Republic.

Data analysis confirmed that individuals with overweight have a significantly higher risk of developing hypercholesterolemia, aligning with the well-documented association between obesity and dyslipidemia^3,10^. These findings underscore the importance of weight management and maintaining a normal BMI as strategies for the prevention of hypercholesterolemia and, consequently, cardiovascular diseases.

In addition, our research unveiled ethnic disparities in cholesterol levels and the incidence of hypercholesterolemia. These discrepancies among ethnic groups could potentially be influenced by factors like genetic predispositions^18^, cultural dietary habits^19^, lifestyle choices, or socio-economic aspects^20^. Our findings were consistent with other studies with Kazakhstani population^21^.

Despite significant associations found between hypercholesterolemia and specific demographic characteristics, the results underscore the multifactorial nature of this condition. The complex interplay of genetic, metabolic, dietary, physical, and socio-economic factors necessitates a comprehensive approach to the prevention and management of hypercholesterolemia^13,22^.

This implies not only individualized medical interventions but also broad public health strategies aimed at improving dietary quality, promoting physical activity, enhancing the population’s educational level in health matters, and ensuring equal access to quality medical care.

The current research has successfully achieved its objectives, providing crucial insights into the scale of hypercholesterolemia problem in Kazakhstan and revealing the differences among distinct population groups. The findings offer a solid foundation for future research and can be used to guide public health policy in tackling hypercholesterolemia, with the ultimate goal of reducing the burden of cardiovascular disease in the Republic of Kazakhstan.

## Conclusion

Our findings affirm the necessity of developing and implementing targeted, scientifically substantiated strategies for the prevention and management of hypercholesterolemia in Kazakhstan. Given the multifactorial nature of this condition, effective strategies should encompass both individualized medical interventions and public initiatives aimed at enhancing the overall health of the population.

### Prospects for future research

Further research is required to elucidate the mechanisms linking the identified risk factors with hypercholesterolemia and to develop more effective prevention and treatment strategies. A significant direction for future studies involves exploring the genetic predispositions to hypercholesterolemia across different ethnic and demographic groups, as well as theroscl the impact of socio-economic conditions and lifestyle on the development of this condition. Additionally, the effect of long-term lifestyle and dietary changes on the dynamics of cholesterol levels in the population warrants longitudinal studies.

### Study strengths and limitations

The study’s robust sample size lends credibility to its findings. The multi-stage cluster sampling method and consideration of various demographic groups ensure a broad representation. The detailed description of the study design, including ethical considerations, sampling method, covariates, and statistical analysis, provides transparency.

Nonetheless, this study has some limitations. Given the cross-sectional design, it provides a snapshot of the situation at a specific point in time and does not allow for causality inference.

Detailed lipid profiling, including LDL and HDL cholesterol, was not conducted due to the resource-intensive nature of such analyses in a large-scale study.

Additionally, although the large sample size and multi-stage cluster sampling method strengthen the validity of our findings, some regions or ethnic groups might be underrepresented.

## Methods

### Ethical considerations

The S.D. Asfendiyarov Kazakh National Medical University Local Ethics Committee approved the study, as documented in Protocol No. 12 (118) dated September 28, 2021. Additionally, this research was registered at ClinicalTrials.gov under the identifier NCT05122832. All methods were performed in accordance with the relevant guidelines. Informed consent was obtained from all subjects and for uneducated participants, informed consent was obtained from their guardian/legally authorized representative. Research had been performed in accordance with the Declaration of Helsinki.

### Study design and sample

Our study employed a cross-sectional design and focused on the adult population of Kazakhstan from October 2021 to May 2022. This was a nationally representative survey. A total of 6720 individuals aged between 18 and 69 were voluntarily recruited across all 17 regions of the country, including the cities of Almaty, Astana, and Shymkent. Participants recruited from October 2021 and May 2022.

### Study context

Kazakhstan, located in Central Asia, is administratively subdivided into 14 regions, three cities of republican significance (Astana, Almaty, and Shymkent), and 177 districts. The majority of the country’s 20 million inhabitants live in urban areas, despite its low population density of 6 people per square theroscl. (https://stat.gov.kz/).

### Sampling

In this study, we employed a weighted multistage cluster sampling approach, segregating participants into eight cohorts, further bifurcated into four age brackets: 18–29 years, 30–44 years, 45–59 years, and 60–69 years. This stratification was also conducted by gender, ensuring representation of both males and females within each age cohort. The sample size was ascertained using the World Health Organization’s designated STEPS tool in the format of an Excel-based sample size calculator. The parameters utilized were:

Confidence interval of 95% with a Z-score of 1.96;

Hypothesized prevalence of risk factors at 0.5;

Standard error of 0.05;

Design effect coefficient of 1.5;

Projected response rate at 70%.

Subsequent computations yielded a requisite sample size of n = 6585.

The employed multistage cluster sampling encompassed three tiers, with clusters established at every stage. Initially, primary sampling units were delineated, encompassing districts and urban centers. These units were proportionally selected from all economic regions, sourcing data from the Bureau of National Statistics and the Agency for Strategic Planning and Reforms of the Republic of Kazakhstan.

In the secondary stage, Primary Health Care (PHC) facilities serving the local populace were identified as the secondary sampling units (SSUs). Data for this selection was procured from the Republican Centre for Healthcare Development, a subsidiary of the Ministry of Health of the Republic of Kazakhstan. The register of PHCs was accessed, revealing the patient population of each facility. The SSUs were selected via a random sampling procedure, proportionate to the patient population of each PHC.

For the tertiary stage, households and individual respondents were the focal sampling units. The household quota per PHC was derived using the equation:

Household per PHC = 6585/240 ≈ 28.

Subsequent calculations yielded a final aggregate sample size of 6720. To curate the household sample, a roster of households under the purview of the selected PHCs was compiled. Households were chosen randomly from each facility utilizing the Randhold.xls tool for study inclusion. The final respondent selection, comprising individuals aged 18–69 from the chosen households, was executed employing the Kish methodology. This technique encompassed a stratified random selection based on the gender and age demographics of eligible household members.

The research noted a commendable participation rate of 95%. This elevated engagement can be ascribed to comprehensive participant awareness regarding the research objectives and its inherent importance. The incorporation of trusted local laboratories potentially augmented this rate.

### Covariates

Based on the presented tables, the covariates in this study included age, sex, geographical location (region), and ethnicity. For each covariate, we computed variables related to cholesterol levels, including average cholesterol levels, the percentage of individuals with cholesterol above 5.0 mmol/l. We used WHO criteria defined raised cholesterol as total cholesterol ≥ 5.0 mmol/l or 190 mg/dl^23^. These computations were carried out separately for males and females, and then for the overall population.

### Laboratory techniques

The study relied on the analysis of blood biochemical markers. Specifically, we measured total cholesterol levels and identified the proportion of individuals with hypercholesterolemia, defined as cholesterol levels exceeding the WHO recommended threshold of 5.0 mmol/l (https://www.who.int/data/gho/indicator-metadata-registry/imr-details/3236). Our investigation focused on individuals with cholesterol levels above 5.0 mmol/l.

### Statistical analysis

Statistical computations were performed using SAS OnDemand for Academics (release 3.81, Carry, NC, USA). We presented categorical variables as frequency and percentage, and continuous variables as mean and standard deviation (SD).

To assess the normality of continuous variables, we employed the Kolmogorov–Smirnov test. We used the one-way analysis of variance for analysis and the Kruskal–Wallis test for non-normally distributed continuous variables, which were presented as median (interquartile range).

For each association, odds ratios (Ors) with 95% confidence intervals (Cis) were calculated to estimate the strength and direction of the relationships.

Initially, univariate logistic regression analyses were conducted to evaluate the crude associations between each predictor variable and hypercholesterolemia. Subsequently, multivariate logistic regression analysis was performed to adjust for potential confounders and to determine the independent effect of each predictor on hypercholesterolemia risk.

All data were weighted according to the sex and age distribution of the Kazakhstan population. Statistical significance was set at an alpha level of 0.05.

## Data Availability

All available data was indicated within manuscript text.
